# Sample size calculation to externally validate scoring systems based on logistic regression models

**DOI:** 10.1371/journal.pone.0176726

**Published:** 2017-05-01

**Authors:** Antonio Palazón-Bru, David Manuel Folgado-de la Rosa, Ernesto Cortés-Castell, María Teresa López-Cascales, Vicente Francisco Gil-Guillén

**Affiliations:** 1 Department of Clinical Medicine, Miguel Hernández University, San Juan de Alicante, Alicante, Spain; 2 Department of Pharmacology, Pediatrics and Organic Chemistry, Miguel Hernández University, San Juan de Alicante, Alicante, Spain; 3 Department of Molecular Neurobiology, Neurosciences Institute (Miguel Hernández University and Consejo Superior de Investigaciones Científicas), San Juan de Alicante, Alicante, Spain; Iranian Institute for Health Sciences Research, ISLAMIC REPUBLIC OF IRAN

## Abstract

**Background:**

A sample size containing at least 100 events and 100 non-events has been suggested to validate a predictive model, regardless of the model being validated and that certain factors can influence calibration of the predictive model (discrimination, parameterization and incidence). Scoring systems based on binary logistic regression models are a specific type of predictive model.

**Objective:**

The aim of this study was to develop an algorithm to determine the sample size for validating a scoring system based on a binary logistic regression model and to apply it to a case study.

**Methods:**

The algorithm was based on bootstrap samples in which the area under the ROC curve, the observed event probabilities through smooth curves, and a measure to determine the lack of calibration (estimated calibration index) were calculated. To illustrate its use for interested researchers, the algorithm was applied to a scoring system, based on a binary logistic regression model, to determine mortality in intensive care units.

**Results:**

In the case study provided, the algorithm obtained a sample size with 69 events, which is lower than the value suggested in the literature.

**Conclusion:**

An algorithm is provided for finding the appropriate sample size to validate scoring systems based on binary logistic regression models. This could be applied to determine the sample size in other similar cases.

## Introduction

The predictive model most widely used in medicine to determine the onset of a clinical event (disease, relapse, death, healing…) is the binary logistic regression model. The probability of an event based on a series of parameters (explanatory variables) is obtained through a closed formula including addition, multiplication and exponentials [1]. Consequently, we are unable to determine this probability without the use of an electronic device. For this reason, researchers from the Framingham Heart Study developed an algorithm that adapted these mathematical models for use in routine clinical practice without the need for electronic devices, using scoring systems [2].

The algorithm begins by categorizing all the explanatory variables, associating each category with a score obtained through weighting the model coefficients. This then gives a finite set of total scores which: 1) is determined by the sum of all the scores associated with each of the explanatory variables, and 2) has an associated event probability [2]. In other words, the algorithm transforms a multivariate binary logistic regression model into another with a single explanatory variable (total score), which has a finite number of values, allowing the event probability to be calculated previously for each score.

Both the logistic regression models and the particular case of scoring systems must be validated externally for use in other populations. To carry out this process, both discrimination and calibration must be examined [3]. Discrimination consists of determining whether a higher event probability predicted by the model can differentiate between those subjects who experience an event and those who do not. To address this question, the area under the receiver operating characteristic (ROC) curve (AUC) is calculated [4]. Calibration involves analyzing whether the event probabilities predicted by the model correspond to those observed in reality. Generally, this process has been evaluated by categorizing into risk groups and through the logistic recalibration framework with a linear predictor [5]. However, it is preferable and advisable to use smooth calibration plots based on linear splines or loess [5,6].

When any study requiring statistical tests is performed, such as the external validation of a predictive model, it is necessary to calculate the number of subjects needed to accurately conclude that the results obtained in the sample can be extrapolated to the study population [7]. Most studies undertaken in clinical research, such as estimating the AUC [4], have a closed formula for obtaining the sample size based on a set of parameters (expected population values, type I and type II error, ratio between samples…). However, the determination of the calibration of a predictive model does not have a closed formula. For this reason, simulation studies have been performed to ascertain how many patients are needed to be able to say that the predictive model is well calibrated [5,8]. These studies have concluded that it takes at least 100 events and 100 non-events, regardless of the predictive model being addressed [5,8]. However, when approaching the problem of calculating sample size, factors exist that influence the calibration plot, such as model parameterization [5], incidence of the event being assessed, and the discrimination of the predictive model (AUC) [9]. In other words, we should not establish a single value (100 events and 100 non-events) to check the calibration of all predictive models.

Considering the usefulness of scoring systems in medicine (concrete case of logistic regression models) the fact that there is just one single sample size to validate any predictive model (despite the influence of different factors and that data collection may be laborious) means it is necessary to optimize the sample size so that it can efficiently validate a scoring system statistically without having to collect an excessive number of patients.

The objective of this paper is to explain an algorithm to determine the number of subjects to externally validate a scoring system based on a logistic regression model, which is a particular type of predictive model with a single linear predictor. In other words, we are determining the sample size calculation to externally validate a scoring system of the detailed characteristics. To illustrate how to use this algorithm, it will be applied to an already published scoring system that assesses mortality in intensive care units (ICU) [10]. To address these issues we will adhere to the following structure: first, a synthesis of the concepts of calibration by smooth curves and the AUC, followed by details of the suggested algorithm (sample size calculation). This algorithm will then be applied to the scoring system for mortality in the ICU and finally, a methodological discussion of the proposed algorithm will be provided.

## Materials and methods

### The area under the receiver operating characteristic curve

Suppose we have a random sample of *n* subjects {1,2,…,*i*,…,*n*}, where for each subject we have collected two random variables *x*_*i*_ and *z*_*i*_, where *x* is a quantitative variable (discrete or continuous) and *z* an event indicator variable, i.e., it takes the value 1 when a subject has experienced the event and 0 when a subject has not. Our goal is to determine whether the variable *x* can discriminate (differentiate or distinguish) between subjects who experience an event and those who do not; that is, if higher values of *x* are associated with an increased event probability. Note that this could be done in the opposite way; i.e., smaller values of *x* associated with an increased event risk. Without loss of generality, we will proceed using the first method, as we can move from the second to the first case by multiplying the variable *x* by −1.

We define the sets: *E* = {*i*: *z*_*i*_ = 1, *i* = 1,…,*n*} and $\bar{E}=\{i\;:\;z_{i}=0,i=1,\ldots ,n\}$, equivalent to subjects who have experienced an event and those who have not, respectively. Note that $E\cup \bar{E}=\{1,\ldots ,n\}$ and $E\cap \bar{E}=\emptyset$. With all these elements we are able to define the ROC curve [4], which is obtained by joining the following points on a Cartesian graph restricted to [0,1] *x* [0,1]:

$$\left(1-\frac{\left|i\in \bar{E}\;:\;x_{i}<x\right|}{\left|\bar{E}\right|},\frac{\left|i\in E\;:\;x_{i}\geq x\right|}{\left|E\right|}\right)\;x\in \{x_{1},x_{2},\ldots ,x_{i},\ldots ,x_{n}\},$$

with |·| being the cardinal function of a given set, i.e., the number of elements contained in said set. For any value $\tilde{x}$ of the random variable *x*, the two components of each point on the Cartesian graph correspond respectively to 1-specificity and the sensitivity of a diagnostic test in which positive is defined as $x\geq \tilde{x}$ and negative as $x<\tilde{x}$ [11].

To calculate the area under the curve in the space [0,1] *x* [0,1] (AUC), assume two subjects *j* ∈ *E* and $l\in \bar{E}$. Now we define:

$$S(j,l)=\left\{ \begin{array}{cc} 1 & if\;x_{j}>x_{l}\\ 1/2 & if\;x_{j}=x_{l}\\ 0 & if\;x_{j}<x_{l} \end{array}\right..$$

The calculation of the AUC is obtained through [4]:

$$AUC=\frac{1}{\left|E|\cdot |\bar{E}\right|}\cdot \sum_{j\in E}\sum_{l\in \bar{E}}S(j,l).$$

Note that if *x* is a continuous variable *S*(*j*,*l*) it will never take the value of 1/2.

The AUC is a way to measure the discrimination of a quantitative variable regarding the occurrence of an event. Its interpretation is the following: the closer the AUC is to one indicates that the variable *x* discriminates to a higher degree which subject has experienced an event [4].

We are now interested in determining an $\hat{x}$ value of the variable *x* (cut-off point) to distinguish with minimal error between subjects with and without an event, i.e., consider positive (subject with event) if $x\geq \hat{x}$ and negative in the opposite case (subject without event). The literature on ROC curves uses that value of the random variable *x* that minimizes $\sqrt{{\left(1-Sensitivity(x)\right)}^{2}+{\left(1-Specificity(x)\right)}^{2}}$ [11].

### Scoring systems based on logistic regression models

A scoring system is defined by the following elements [2]: 1) A set of possible score values (consecutive integers): {*x*_*min*_,*x*_*min*_ + 1,…,−1,0,1…,*x*_*max*_ − 1,*x*_*max*_}, where *x*_*min*_ and *x*_*max*_ represent the minimum and maximum score of the system, respectively. We now denote *x* as the score variable, which has *x*_*max*_ − *x*_*min*_ + 1 possible values.

2) A binary logistic regression model defined as *logit*(*z*) = *β*_0_ + *β*_1_ · *x*, with *z* being the indicator variable of the event and *β*_0_ and *β*_1_ the model coefficients associated with the constant and the varying score, respectively. Through these parameters (*β*_0_ and *β*_1_) we can obtain the random variable event probability *p* for each score *x* by the expression 11+exp⁡(−(β0+β1·x)). Note that since *x* has a finite number of values, *p* will too.

### Smooth calibration for the scoring system

Take a random sample of *n* subjects {1,2,…,*i*,…,*n*} where for each subject *i* we have *x*_*i*_ (the value of the score on a scoring system as defined above) and *z*_*i*_ (taking the value 1 if the subject has experienced an event and 0 otherwise). In turn, since we are using a scoring system, we have (using the above notation) *x*_*min*_,*x*_*max*_,*β*_0_ and *β*_1_, and in consequence *p*_*i*_ (probability of event).

For each subject *i* we now define the random variable *L*_*i*_ = *β*_0_ + *β*_1_ · *x*_*i*_. Smooth calibration consists of fitting a logistic regression model to the set {(*z*_*i*_,*L*_*i*_),*i* = 1,…,*n*} with the parameterization *logit*(*z*) = *a* + *f*(*L*), where *f* is a smooth function of *L*, like splines or loess transformations, and a is the intercept of the model [5]. Through this new model we obtain the observed probabilities of the event and compare them with those predicted by the scoring system through a Cartesian graph. This graph will be represented in the space [0,1] *x* [0,1] and the straight line joining the points (0,0) and (1,1) will be added, as it represents the observed probabilities corresponding to those predicted by the scoring system. The smooth curve will be represented together with its associated confidence intervals, which can be obtained through bootstrapping [5]. Note that our system will have a total of *x*_*max*_ − *x*_*min*_ + 1 points represented on the Cartesian graph.

### The estimated calibration index

The estimated calibration index (ECI) is a measure that has been proposed to determine the lack of calibration of a predictive model [5,12]. The ECI consists of calculating the mean squared difference between the observed risk (obtained by smooth curves) and the risk predicted by the model in a total of *N* observations (by bootstrapping it would be in each of the samples). The ECI has a range of values from 0 to 100, where the null value corresponds to absolute perfection between the model and reality [5]. Although the ECI summarizes the lack of calibration in a single number, it has been observed that small values thereof (ECI = 1.67) produce models that are not well calibrated [12]. In other words, if a model is well calibrated, it will have a low ECI value, but the opposite does not hold true. In short, it is a necessary but not sufficient condition. Consequently, we have to represent the Cartesian graph of the models that obtain a low ECI.

### The proposed algorithm to calculate the sample size to externally validate a scoring system

Using all the concepts defined above (AUC, scoring systems, smooth calibration and the ECI) we now detail an algorithm to evaluate how to calculate the sample size to externally validate a scoring system based on a logistic regression model, since we may have a sample size with a number of events and non-events different than 100, as is stated in the literature [5,8]. We must bear in mind that two aspects must be assessed (discrimination and calibration), the first of which does not need an excessive sample size to find statistically significant differences [8]. However, obtaining the sample size to determine if a model is well calibrated requires further study, using simulated samples [8]. For this reason, we will focus on the sample size for smooth calibration.

First, a few considerations; as noted above, the ECI is a measure that can help us with this task, since values close to 0 are necessary, but not sufficient, to say that a model is well calibrated. Therefore, we establish cut-off points near the null value for the ECI and determine if the model is well calibrated through the interpretation of the smooth calibration plot. We must also bear in mind that the random variable of the scores (*x*) can be considered to have a multinomial distribution since it has a finite number of values. In addition, we must have the proportion of subjects with an event (*p*_*event*_), and then establish a possible range of values for the number of events (*n*_*event*_) in order to check its calibration. With these considerations and the concepts discussed above, we can now detail the proposed algorithm:

1. Establish *n*_*event*_ (if it is the first time this step is initiated, *n*_*event*_ takes the minimum value of the possible range of values to check):
  - Simulate a random sample from the vector (*x*,*z*) through the multinomial distribution of the scores and from the logistic regression model associated with the scoring system, with *n*_*event*_ subjects with the event and with $n_{non-event}=\frac{1-p_{event}}{p_{event}}\cdot n_{event}$ subjects without the event. Note that *n*_*non–event*_ could have decimals, so we round it to the nearest whole number.
  - In the sample in step 1a determine the AUC and observed event probabilities for each score through smooth curves.
  - Repeat steps 1a and 1b a predetermined number of times *N* (for example, 1000 times) in order to construct the distribution of these parameters. Once the above steps have been repeated *N* times, continue with step 2.
2. Determine the ECI value with the total *N* observations performed, save the smooth calibration plot with the confidence intervals only and calculate confidence intervals for the AUC. Note that we are only interested in the confidence intervals in order to have a threshold for possible population values, that is, with a high probability of ensuring that the model is well calibrated and can properly discriminate the subject with an event.
3. Recalculate *n*_*event*_ as *n*_*event*_ = *n*_*event*_ + 1 and go to step 1, unless we have already verified the full range of possible values for *n*_*event*_, in which case we go to step 4.
4. With the cut-off points determined a priori for the ECI, create indicator variables to determine whether the number of events *n*_*event*_ verifies that the ECI is lower than these cut-off points.
5. Construct the ROC curves with *n*_*event*_ (quantitative variable) and the indicator variables in the ECI smaller than the cut-off points.
6. Determine the optimum point of *n*_*event*_ for each of the ECI cut-off points, as explained in the section on ROC curves.
7. Interpret the smooth calibration plots of the sample sizes obtained in step 6.
8. Set the sample size as the minimum value of *n*_*event*_ from step 6 that is properly calibrated.

### Case study

We then applied the proposed algorithm to a scoring system for predicting mortality in the ICU [10]. The minimum score of this system is *x*_*min*_ = 0 points and the maximum score is *x*_*max*_ = 15 points, the coefficients of the logistic regression model associated with the system are *β*_0_ = −5.92252114678228 and *β*_1_ = 0.6, the proportion of events is *p*_*event*_ = 0.10781990521327014218009478672986 and the probability distribution for each of the associated scores (ordered from *x*_*min*_ = 0 to *x*_*max*_ = 15) is (0.29023508137432200,0.03887884267631100,0.09222423146473780, 0.18625678119349000,0.05967450271247740,0.08318264014466550, 0.06057866184448460,0.02893309222423150,0.02441229656419530, 0.02622061482820980,0.03345388788426760,0.00994575045207957, 0.03526220614828210,0.02893309222423150,0,0.00180831826401447). These data were obtained from the original publication [10]. The established range of possible values for *n*_*event*_ was between 25 and 1000. The smooth curves were performed using linear splines.

To visualize the influence of sample size on discrimination and calibration, line graphs for the confidence intervals for the AUC and ECI were created. This evolution was analyzed using a video for soft calibration plots, which shows the adjustment to the perfect line of the curves with increasing *n*_*event*_. The cut-off points established for the ECI were 2, 1.75, 1.5, 1.25, 1, 0.75, 0.5 and 0.25.

## Results

**Fig 1:**
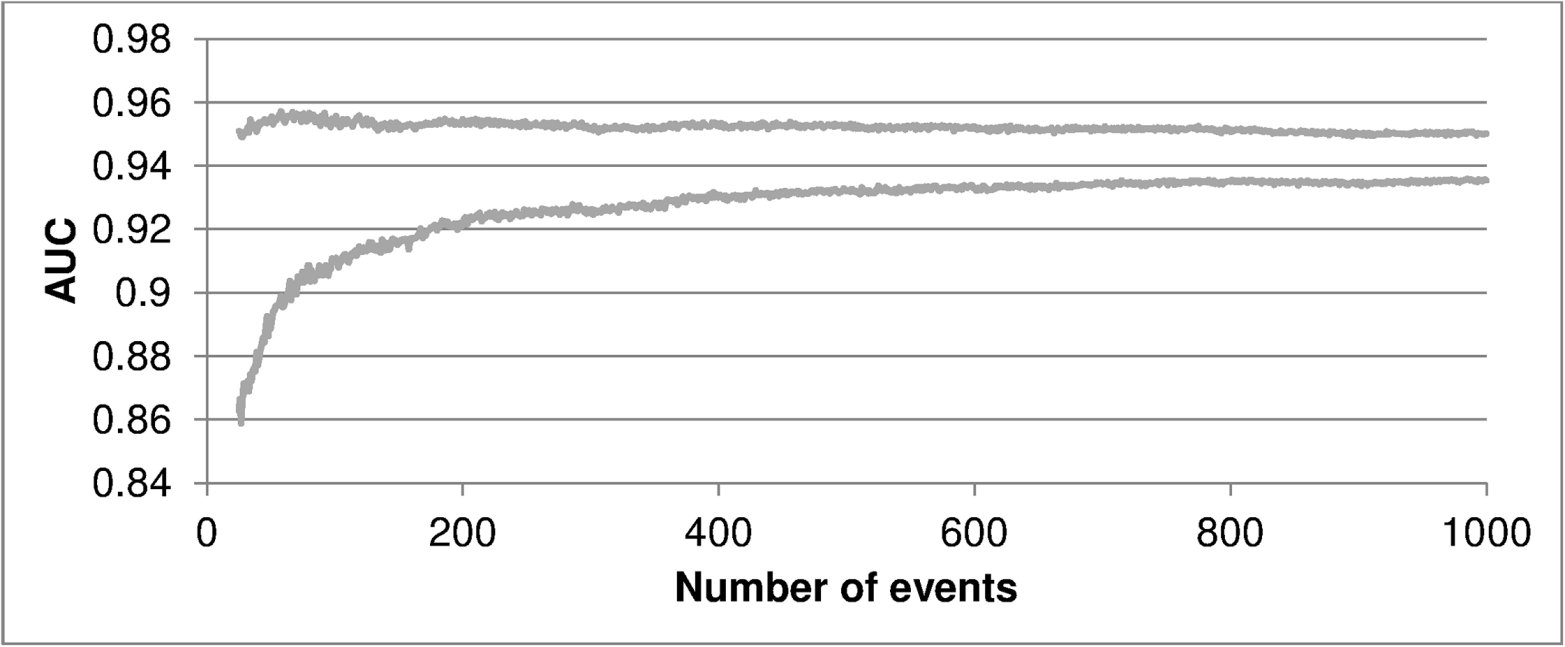
Confidence intervals for the area under the ROC curve according to the number of events in the sample. AUC, area under the ROC curve.

**Fig 2:**
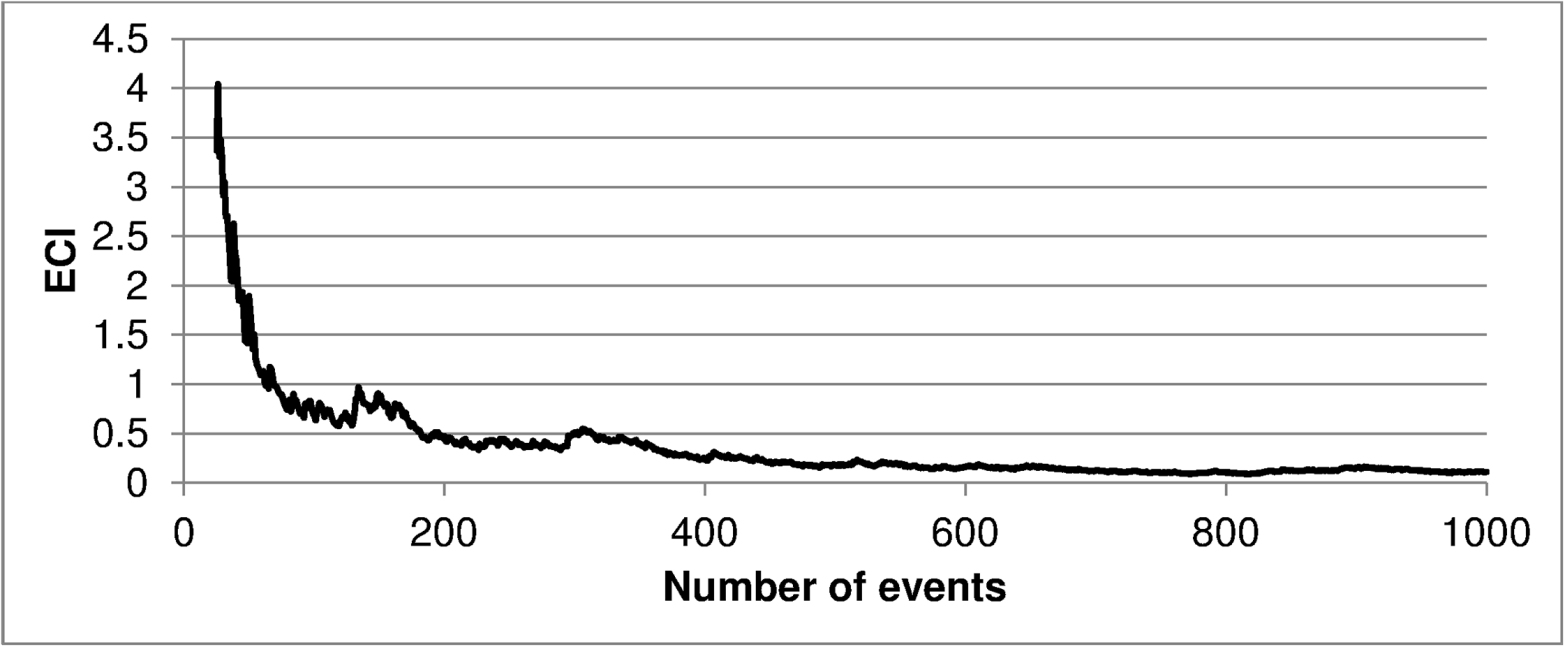
Estimated calibration index values according to the number of events in the sample. ECI, estimated calibration index.

Fig 1 shows the evolution of the AUC as the number of events in the sample increases, while Fig 2 represents the same evolution for the ECI. This evolution for smooth curves can be viewed in S1 Video. As can be seen, by increasing the sample size the errors are reduced and the bars of the smooth curve approach the perfect condition. These charts and the video indicate the presence of a certain point (number of patients) where we have a reduced error to carry out our external validation.

**Fig 3:**
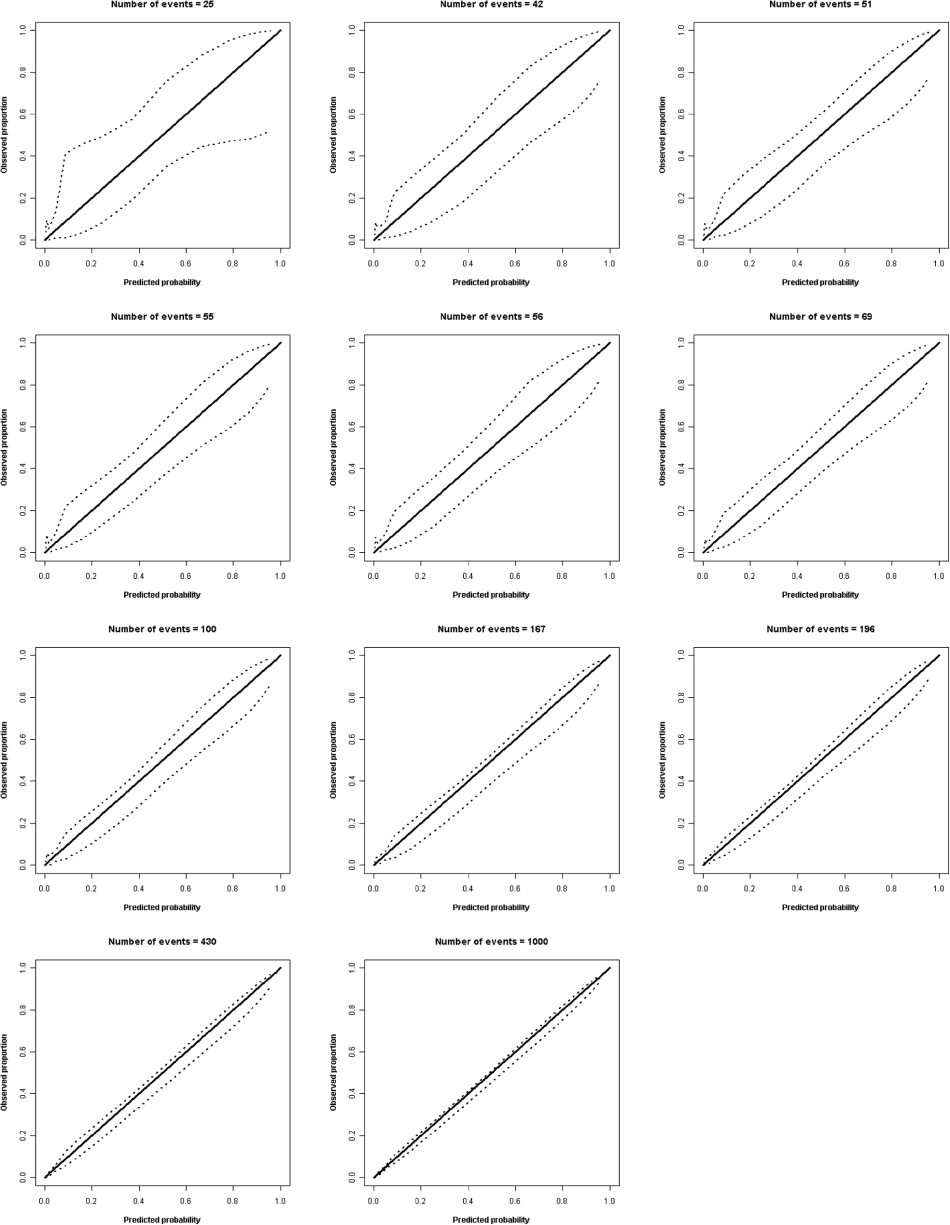
Smooth calibration plots (linear splines) for several sample sizes. The dashed lines denote the confidence intervals. The central line denotes the perfect prediction.

With the cut-off points chosen for the ECI (2, 1.75, 1.5, 1.25, 1, 0.75, 0.5 and 0.25), the number of events for the sample following our algorithm was 42, 51, 55, 56, 69, 167, 196 and 430, respectively. The smooth calibration plots for these sample sizes, along with the initial value of the verified range (*n*_*event*_ = 25), the value suggested in the literature (*n*_*event*_ = 100) and the final value of the range (*n*_*event*_ = 1000), are shown in Fig 3. Note that these images are screenshots from the previous video with sample sizes predetermined by the algorithm; as the sample size increases the bars for the confidence intervals become closer to the perfect condition. Here we see that the minimum number of events that obtain good calibration is 69. This is complemented by an ECI<1.25. If we calculate the total number of patients in the sample through *p*_*event*_, this is 640 patients (69 deceased and 571 living). This sample size would have 100 deceased and 828 living patients (938 in total) as recommended by the literature, representing 298 patients more than by following our algorithm.

## Discussion

Our study developed an algorithm to calculate the sample size to externally validate a scoring system based on a binary logistic regression model, analyzing the smooth calibration plot and lack of calibration of this plot from the calculation of the ECI. As an example, the algorithm was applied to a scoring system to predict mortality in the ICU.

When comparing our algorithm with that published in the scientific literature, we note that other studies have considered a universal point (number of events/non-events = 100) [5,8]. As mentioned above, this is not entirely correct, because according to the predictive model that we are externally validating, the sample size for this validation should be independent [9]. However, we must bear in mind that the algorithm we have developed is for scoring systems, which are a specific case of binary logistic regression models.

Regarding the cut-off points of the ECI, we established this system to determine our sample size. However, another approach to this problem could be to consider all the calibration graphs and visually choose the graph that indicates the scoring system is properly calibrated. We wanted to incorporate the ECI because it is an objective way to measure lack of calibration and, when supplemented by the calibration graph, enabled us to view the issue in a more rigorous manner [5].

We recommend the use of our algorithm to calculate the sample size to externally validate scoring systems based on binary logistic regression models. Its application provides the number of patients required for the study, which may be fewer (or more) than 100 events and 100 non-events, as has been specified in the scientific literature [5,8].

According to the results of the case study performed (mortality in ICU) a sample size of 640 patients was obtained, which included 69 deaths. Consequently, if others plan to conduct studies to externally validate the scoring system to predict mortality in the ICU [10], the sample size calculation for these studies is available to them.

The main strength of this work is the algorithm developed to calculate the sample size to externally validate scoring systems based on binary logistic regression models. This subject has not been addressed in depth in the scientific literature, with the use of 100 events and 100 non-events being the recommendation, regardless of the characteristics of the model [5,8]. The value of 100 should not be fixed, however, because there are factors that have been shown to influence the calibration graph [9]. We also highlight the use of smooth curves rather than risk categorizations such as the Hosmer-Lemeshow test, as they give greater validity to the results [5]. Finally, we believe that this algorithm can be extended to more complex cases, such as scoring systems based on survival models or logistic regression models/overall survival, since scoring systems are a specific case of the same.

As a limitation, we note that this calculation carries a high computational cost due to the necessity of multiple bootstrapping samples for each number of events from the proposed range. In our case, our range had 976 possible values and 1000 simulations in each value, equivalent to a total of 976,000 simulations, in which the AUC and the observed values were calculated through smooth curves. However, if we consider the benefit that the use of this algorithm can provide, this would not be a limitation. In our example we have reduced the sample size suggested by the literature by 298 patients, which corresponds to a substantial reduction in both economic costs and the time needed to recruit study participants. In other words, the algorithm is useful to assess the issue being studied (sample size to validate scoring systems based on binary logistic regression models).

As a new line of research, we propose adapting this algorithm to a scoring system based on survival models. To do this, we will need to set cut-off points for prediction time and obtain the observed event probabilities of these cut-off points through smooth curves. These probabilities will depend on the corresponding value in the scoring system and the baseline survival at the time being assessed [2]. We encourage other authors to adapt our algorithm to general logistic regression models.

## Conclusions

This paper provides an algorithm to determine the sample size for validating scoring systems based on binary logistic regression models. The algorithm is based on bootstrapping and basic concepts when validating a predictive model (ROC curve, smooth calibration plots, and ECI). We applied the algorithm to a case to help readers better understand its application.

## Supporting information

S1 Video Smooth calibration plots for the example (number of events from 25 to 1000). (MP4)
